# Food for Soul—Older Immigrants’ Food Habits and Meal Preferences After Immigration: A Systematic Literature Review

**DOI:** 10.1007/s10903-023-01571-5

**Published:** 2024-01-02

**Authors:** Daniela Lillekroken, Asta Bye, Liv Halvorsrud, Laura Terragni, Jonas Debesay

**Affiliations:** 1 Department of Nursing and Health Promotion, Oslo Metropolitan University, PB 4, St. Olavs Plass, N-0130 Oslo, Norway; 2 European Palliative Care Research Centre (PRC), Department of Oncology, Oslo University Hospital, and Institute of Clinical Medicine, University of Oslo, Oslo, Norway

**Keywords:** Dietary acculturation, Food habits, Healthcare services, Older immigrants, Meal preferences, Systematic review

## Abstract

There are few primary studies that focused on the older immigrants’ food habits and meal preferences after immigration and settlement in a new country. A comprehensive database search for literature was conducted in May 2021 and upgraded in September 2021. Ten databases (Medline (Ovid), EMBASE (Ovid), PsycInfo (Ovid), Cinahl (EBSCOhost), Food Science Source (EBSCOhost), SocIndex (EBSCOhost), Social Care Online, Applied Social Sciences Index & Abstracts (ASSIA), Web of Science and Google Scholar), were scanned for original, peer-reviewed papers published in English. The review was conducted and reported in accordance with the PRISMA 2020 guidelines and SWiM items. Out of 3069 records, 10 papers were included for thematic synthesis. A data synthesis across all studies resulted in three main findings: (i) the significance of food in maintaining cultural identity, (ii) the continuity of traditional food culture and (iii) adapting to the host country’s food culture. Although different forms of dietary acculturation occur throughout life, older immigrants often want to maintain their traditional food habits and meal preferences. For them, traditional eating habits offer comfort and security by serving as a means of identifying who they are and reminding them where they have come from. Public health and social services play an important role in providing cultural nutritional care to older immigrants; therefore, this issue should be carefully addressed by professionals and future research.

*Registration*: The review protocol was registered in the International Prospective Register of Systematic Reviews (PROSPERO) on 27 September 2022 with registration number CRD42022358235.

**Supplementary Information:**

The online version contains supplementary material available at 10.1007/s10903-023-01571-5.

## Introduction

Global population migration has changed the demographics of many countries leading to culturally diverse societies [1]. In 2020, 281 million people resided in a country that was not their country of birth [2]. The world’s population is aging rapidly, and older adults compose a larger proportion of the world’s population [3]. Thus, the combination of migration and an ageing population are two major challenges that many countries face in the twenty-first century [4]. World Health Organization [3] defines older people as persons who are over 60 years of age. Older immigrants are defined as people aged 60 years or older, who have moved away from their place of usual residence to live temporarily or permanently in a foreign country [2]. Older people who receive healthcare services are facing challenges related to inadequate nutritional care and malnutrition [5]. Nutritional challenges is also a concern among older immigrants; therefore, to deal with the challenges related to nutrition, it is important that healthcare professionals understand the nutritional needs of the ageing population, as well as their preferences and perceptions related to food.

Because of the differences in cultural, religious, and socioeconomic backgrounds, immigrants may have food habits and meal preferences other than those of the host population. This is particularly pertinent for those who are in vulnerable conditions, such as illness and frailty, where immigrants need assistance with food and meals from healthcare services [6]. Results from a scoping review indicate that ethnic food consumption can have a positive impact on mental health among immigrants [7]. Food is not only important for health and in physical sustenance, but it is also linked to an individual’s personality, cultural identity, social practices, and religious beliefs, in addition to generating enjoyment and a supporting quality of life. Moreover, results from a previous study conducted among British Bangladeshis has shown that behaviors surrounding the preparation and consumption of ethnic foods are some of the most resilient aspects of a migrant culture [8].

After immigration and settlement in a new country, immigrants are exposed to the host country, resulting in physical, biological, political, economic, sociocultural and psychological changes [9]. The process through which immigrants and their children are exposed to the values, behavioural norms and attitudes of the host society is defined as acculturation [10] (p. 708). The acculturation process also includes food consumption [11], which is often called dietary acculturation. Dietary acculturation involves both the retention of certain dietary behaviours and incorporation of new foods and eating habits from the new country [12]. The results from a study conducted by a Romanian research team revealed that the longer Romanian immigrants resided in Andalucía, Spain, the more they perceived changes in their food consumption habits, thus demonstrating a gradual adoption of Spanish foods [13]. After acculturation has occurred, the most common feature is so-called ‘bicultural food habits’, indicating the consumption of traditional food or traditional meals while adding the host population’s food and meals [12].

Although immigrant groups tend to adopt the food habits of the host country [12], the opportunity to have access to traditional food and preserve familiar meal patterns is deemed important [14]. Food communicates history, memory, feelings, and social status [15]. Moreover, food is a source of comfort in life [16] and central to all the processes needed to adapt to a host country [17]. Meals are opportunities for socialisation and interaction, both of which are important for a person’s identity and integrity [18]. Although the meaning of meals and food has evolved across the stages of the life cycle, the eating habits of elderly individuals tend to be characterised by lifelong patterns of eating that have meaning and significance in their everyday lives [19].

During the ageing process, older immigrants may increasingly become more dependent on others to provide their meals. Traditionally, care for the elderly from minority groups was provided by family members [20]. However, because of the socioeconomic transformations in the host countries and societies at large, family caregiving attitudes have changed, and family caregiving is now somewhat weaking [21]. The responsibility of caring for older family members from immigrant groups, including the provision of nutritional care, has gradually been transferred to public healthcare services [21]. Therefore, healthcare workers providing nutritional care need to be aware of the food habits and meal preferences of elderly from minority groups [22]. Meals that are adequate from a nutritional perspective but fail to meet cultural, taste or religious preferences may be only partially eaten or not eaten at all, which again can have serious health consequences, particularly for vulnerable groups such as older immigrants. Although there are several studies exploring dietary changes after migration and settlement in a new country, there is a paucity of specific knowledge about food habits and meal preferences among older immigrants; therefore, more knowledge is needed. The aim of this systematic review is to identify and synthesise the research studies that address older immigrants’ food habits and meal preferences after immigration and settlement in the host country. The present systematic review will therefore answer the following research question: ‘What are the older immigrants’ food habits and meal preferences after immigration and settlement in the host country?’.

## Methods

The review protocol was registered in the International Prospective Register of Systematic Reviews (PROSPERO) on 27 September 2022 with registration number CRD42022358235.

A systematic review of the literature with qualitative and quantitative study designs was conducted following the recommendations of the Preferred Reporting Items Systematic Review and Meta-Analysis guidelines (PRISMA) [23] and the synthesis without meta-analyses items (SWiM) [24] (Supplementary Files S1&S2).

As a systematic review, the present study does not need ethical approval according to the Norwegian Research Ethics Act. The studies included in the review had ethical approval or clear ethical statements.

### Data Sources and Search Strategy

A comprehensive database search was conducted by three academic librarians during May 2021 and upgraded in September 2021. Three groups of relevant search terms (‘Food’, ‘Immigrants’, ‘Older people’) and their synonyms were used and combined using the Boolean operators AND and OR as per the example search strategy shown in Table 1. Search terms were applied consistently across nine databases, including Medline (Ovid), EMBASE (Ovid), PsycInfo (Ovid), CINAHL (EBSCOhost), Food Science Source (EBSCOhost), SocIndex (EBSCOhost), Social Care Online, Applied Social Sciences Index & Abstracts (ASSIA), Web of Science and Google Scholar.

**Table 1:** Examples of literature search in databases

Database: Ovid MEDLINE(R) and Epub Ahead of Print, In-Process, In-Data-Review & Other Non-Indexed Citations and Daily 1946 to May 11, 2021 Dato:12.05.21 Antall treff: 1635		
#	Searches	Results
1	Eating/ or Diet/	214,140
2	Feeding Behavior/	85,628
3	fasting/ or food preferences/	50,940
4	Food/	34,244
5	exp Meals/	6532
6	Cooking/	12,831
7	(food* or eat* or diet* or nutrit* or meal* or cooking or dining).tw,kw,kf	1,295,342
8	or/1–7	1,398,886
9	"emigrants and immigrants"/ or undocumented immigrants/	13,304
10	Refugees/	10,906
11	"Emigration and Immigration"/	25,451
12	Human Migration/	1295
13	Minority Groups/	14,749
14	Ethnic Groups/ or cultural diversity/	74,539
15	(immigra* or migrant* or ethnic* or racial* or minorit* or refugee* or non western*).tw,kw,kf	286,704
16	or/9–15	347,897
17	aged/	3,195,187
18	"aged, 80 and over"/	958,793
19	frail elderly/	12,400
20	geriatric nursing/	13,672
21	geriatrics/	30,459
22	Health Services for the Aged/	17,987
23	Dementia/	53,503
24	homes for the aged/ or nursing homes/	41,208
25	senior centers/ or adult day care centers/	207
26	((aged or old*) adj2 (people or women or person* or men or immigrant*)).tw,kw,kf	188,204
27	(senior* or geriatric* or elder* or dementia or nursing home*).tw,kw,kf	480,384
28	old*.ti,kw,kf	232,405
29	or/17–28	3,647,617
30	8 and 16 and 29	6681
31	cross-cultural comparison/ or cultural characteristics/ or cultural diversity/	52,707
32	culture/ or acculturation/ or ceremonial behavior/ or food preferences/	55,870
33	Self Concept/	58,058
34	"Quality of Life"/ or personal satisfaction/	225,504
35	(assimila* or accultura* or integrat* or cultural* or culture* or identit* or sociocultural* or multicultural*).tw,kw,kf	1,893,761
36	(ritual* or tradition*).tw,kw,kf	422,225
37	((meal* or food or eating) adj2 (meaning* or habit* or prefer* or context* or practice*)).tw,kw,kf	16,667
38	(quality of life or life quality).tw,kw,kf	311,690
39	(wellbeing* or well being* or wellness).tw,kw,kf	113,628
40	((life or personal*) adj satisf*).tw,kw,kf	9282
41	or/31–40	2,793,174
42	30 and 41	1547
43	(food* or eat* or diet* or nutrit* or meal* or cooking or dining).ti,kw,kf	523,888
44	(immigrant* or migrant* or ethnic* or racial* or minorit* or refugee* or non western*).ti,kw,kf	86,701
45	(senior* or geriatric* or elder* or dementia or nursing home* or old* or aged).ti,kw,kf	523,923
46	43 and 44 and 45	131
47	42 or 46	1635

Database: Embase 1974 to 2021 May 11 Dato:12.05.21 Results: 1923		
#	Searches	Results
1	eating/ or food intake/	167,156
2	eating habit/ or feeding behavior/ or food preference/	107,826
3	eating/ or diet/	260,201
4	food/	74,460
5	meal/	19,616
6	nutrition/	110,997
7	cooking/	17,580
8	(food* or eat* or diet* or nutrit* or meal* or cooking or dining).tw,kw	1,615,941
9	or/1–8	1,741,070
10	immigrant/ or migrant/ or emigrant/	26,926
11	refugee/	13,914
12	undocumented immigrant/	520
13	immigration/ or migration/	46,460
14	Minority group/	15,637
15	Ethnic group/	71,703
16	"ethnic or racial aspects"/ or ethnic difference/ or ethnicity/ or race/ or race difference/ or cultural diversity/	228,971
17	(immigra* or migrant* or ethnic* or racial* or minorit* or refugee* or non western*).tw,kw	384,093
18	or/10–17	552,882
19	aged/ or frail elderly/ or institutionalized elderly/ or very elderly/	3,185,678
20	geriatric patient/	26,051
21	geriatrics/	31,261
22	dementia/	124,095
23	nursing home/	54,844
24	senior center/	406
25	((aged or old*) adj2 (people or women or person* or men or immigrant*)).tw,kw	248,915
26	(senior* or geriatric* or elder* or dementia or nursing home*).tw,kw	671,773
27	old*.ti,kw	276,595
28	or/19–27	3,743,920
29	9 and 18 and 28	9302
30	cultural anthropology/ or cultural diversity/ or eating habit/ or food preference/	82,103
31	cultural factor/	63,868
32	cultural value/	1843
33	identity/ or self concept/	116,519
34	"quality of life"/ or life satisfaction/	519,750
35	wellbeing/ or psychological well-being/	92,492
36	(assimila* or accultura* or integrat* or cultural* or culture* or identit* or sociocultural* or multicultural*).tw,kw	2,313,619
37	(ritual* or tradition*).tw,kw	557,663
38	((meal* or food or eating) adj2 (meaning* or habit* or prefer* or context* or practice*)).tw,kw	23,140
39	(quality of life or life quality).tw,kw	502,526
40	(wellbeing* or well being* or wellness).tw,kw	148,975
41	((life or personal*) adj satisf*).tw,kw	11,400
42	or/30–41	3,659,178
43	29 and 42	2062
44	(food* or eat* or diet* or nutrit* or meal* or cooking or dining).ti,kw	618,836
45	(immigrant* or migrant* or ethnic* or racial* or minorit* or refugee* or non western*).ti,kw	105,783
46	(senior* or geriatric* or elder* or dementia or nursing home* or old* or aged).ti,kw	653,386
47	44 and 45 and 46	165
48	43 or 47	2175
49	limit 48 to conference abstracts	252
50	48 not 49	1923

Database: EBSCOhost Food Science Source Dato:12.05.21 Results: 242		
#	Query	Results
S1	DE "FOOD habits" OR DE "DIET" OR DE "FOOD" OR DE "NUTRITION" OR DE "NUTRITION – Psychological aspects"	79,077
S2	DE "FASTING" OR DE "MEAL frequency"	1,793
S3	DE "FOOD preferences in old age" OR DE "FOOD preferences" OR DE "FOOD & culture"	3,186
S4	DE "COOKING" OR DE "MEALS" OR DE "MEALS – Religious aspects"	28,041
S5	TI ( food* or eat* or diet* or nutrit* or meal* or cooking or dining) OR AB ( food* or eat* or diet* or nutrit* or meal* or cooking or dining) OR KW ( food* or eat* or diet* or nutrit* or meal* or cooking or dining) OR SU ( food* or eat* or diet* or nutrit* or meal* or cooking or dining)	693,522
S6	S1 OR S2 OR S3 OR S4 OR S5	694,161
S7	DE "IMMIGRANTS" OR DE "IMMIGRANT men" OR DE "OLDER immigrants" OR DE "PERMANENT residents (Immigrants)" OR DE "UNDOCUMENTED immigrants" OR DE "WOMEN immigrants" OR DE "ASSIMILATION of immigrants" OR DE "EMIGRATION & immigration" OR DE "REFUGEES"	2,949
S8	DE "MINORITIES" OR DE "ATTITUDES of ethnic groups" OR DE "MINORITY older people" OR DE "MINORITY women" OR DE "RACIAL minorities" OR DE "ETHNICITY" OR DE "RACE"	4,186
S9	DE "ETHNIC groups" OR DE "ASSIMILATION of immigrants"	2,562
S10	DE "CULTURAL pluralism"	373
S11	TI ( immigra* or migrant* or ethnic* or racial* or minorit* or refugee* or "non western*") OR AB ( immigra* or migrant* or ethnic* or racial* or minorit* or refugee* or "non western*") OR KW ( immigra* or migrant* or ethnic* or racial* or minorit* or refugee* or "non western*") OR SU ( immigra* or migrant* or ethnic* or racial* or minorit* or refugee* or "non western*")	36,748
S12	S7 OR S8 OR S9 OR S10 OR S11	37,717
S13	DE "OLDER people" OR DE "FRAIL elderly" OR DE "MINORITY older people" OR DE "OLDER immigrants" OR DE "OLD-old" OR DE "OLDER men" OR DE "OLDER patients" OR DE "OLDER refugees" OR DE "OLDER women" OR DE "ADULT day care centers" OR DE "ELDER care" OR DE "GERIATRICS" OR DE "GERONTOLOGY" OR DE "OLD age" OR DE "OLDER people's attitudes"	8,571
S14	DE "DEMENTIA" OR DE "FOOD preferences in old age"	2,658
S15	DE "SENIOR centers"	23
S16	DE "NURSING home patients"	286
S17	TI ( ((aged or old*) N1 (people or women or person* or men or immigrant*))) OR AB ( ((aged or old*) N1 (people or women or person* or men or immigrant*))) OR KW ( ((aged or old*) N1 (people or women or person* or men or immigrant*))) OR SU ( ((aged or old*) N1 (people or women or person* or men or immigrant*)))	25,309
S18	TI ( senior* or geriatric* or elder* or dementia or "nursing home*") OR AB ( senior* or geriatric* or elder* or dementia or "nursing home*") OR KW ( senior* or geriatric* or elder* or dementia or "nursing home*") OR SU ( senior* or geriatric* or elder* or dementia or "nursing home*")	34,243
S19	TI old* OR KW old* OR SU old*	24,037
S20	S13 OR S14 OR S15 OR S16 OR S17 OR S18 OR S19	63,529
S21	S6 AND S12 AND S20	689
S22	DE "CULTURE" OR DE "ACCULTURATION" OR DE "CROSS-cultural communication" OR DE "CULTURAL assumptions" OR DE "CULTURAL pluralism" OR DE "FOOD & culture" OR DE "FOOD habits" OR DE "CULTURAL values" OR DE "FOOD preferences in old age" OR DE "FOOD preferences"	17,179
S23	DE "CULTURAL identity" OR DE "IDENTITY (Psychology)" OR DE "SOCIOCULTURAL factors" OR DE "CULTURAL competence" OR DE "CULTURAL awareness" OR DE "CULTURE conflict" OR DE "MULTICULTURALISM"	1,205
S24	DE "CROSS-cultural differences"	209
S25	DE "SELF-perception"	862
S26	DE "ASSIMILATION (Sociology)" OR DE "ASSIMILATION of immigrants"	88
S27	DE "QUALITY of life" OR DE "WELL-being" OR DE "LIFE satisfaction"	8,597
S28	TI ( assimila* or accultura* or integrat* or cultural* or culture* or identit* or sociocultural* or multicultural*) OR AB ( assimila* or accultura* or integrat* or cultural* or culture* or identit* or sociocultural* or multicultural*) OR KW ( assimila* or accultura* or integrat* or cultural* or culture* or identit* or sociocultural* or multicultural*) OR SU ( assimila* or accultura* or integrat* or cultural* or culture* or identit* or sociocultural* or multicultural*)	218,968
S29	TI ( ritual* or tradition*) OR AB ( ritual* or tradition*) OR KW ( ritual* or tradition*) OR SU ( ritual* or tradition*)	56,966
S30	TI ( ((meal* or food or eating) N1 (meaning* or habit* or prefer* or context* or practice*))) OR AB ( ((meal* or food or eating) N1 (meaning* or habit* or prefer* or context* or practice*))) OR KW ( ((meal* or food or eating) N1 (meaning* or habit* or prefer* or context* or practice*))) OR SU ( ((meal* or food or eating) N1 (meaning* or habit* or prefer* or context* or practice*)))	19,596
S31	TI ( "quality of life" or "life quality") OR AB ( "quality of life" or "life quality") OR KW ( "quality of life" or "life quality") OR SU ( "quality of life" or "life quality")	14,170
S32	TI ( wellbeing* or "well being*" or wellness) OR AB ( wellbeing* or "well being*" or wellness) OR KW ( wellbeing* or "well being*") OR SU ( wellbeing* or "well being*" or wellness)	13,588
S33	TI ( ((life or personal*) N0 satisf*)) OR AB ( ((life or personal*) N0 satisf*)) OR KW ( ((life or personal*) N0 satisf*)) OR SU ( ((life or personal*) N0 satisf*))	660
S34	S22 OR S23 OR S24 OR S25 OR S26 OR S27 OR S28 OR S29 OR S30 OR S31 OR S32 OR S33	308,240
S35	S21 AND S34	184
S36	TI ( food* or eat* or diet* or nutrit* or meal* or cooking or dining) OR KW ( food* or eat* or diet* or nutrit* or meal* or cooking or dining)	227,639
S37	TI ( immigrant* or migrant* or ethnic* or racial* or minorit* or refugee* or "non western*") OR KW ( immigrant* or migrant* or ethnic* or racial* or minorit* or refugee* or "non western*")	12,705
S38	TI ( senior* or geriatric* or elder* or dementia or nursing home* or old* or aged) OR KW ( senior* or geriatric* or elder* or dementia or nursing home* or old* or aged)	33,636
S39	S36 AND S37 AND S38	78
S40	S35 OR S39	242

Database: APA PsycInfo < 1806 to May Week 1 2021 > Dato:12.05.21 Results: 589		
#	Searches	Results
1	Food/	14,318
2	Food Intake/	14,943
3	Food Preferences/	5312
4	Mealtimes/	847
5	Eating Attitudes/	1628
6	eating behavior/	14,182
7	Food Preparation/	321
8	Diets/	13,518
9	Nutrition/	11,165
10	(food* or eat* or diet* or nutrit* or meal* or cooking or dining).tw	174,296
11	1 or 2 or 3 or 4 or 5 or 6 or 7 or 8 or 9 or 10	178,198
12	Immigration/	23,494
13	Refugees/	6698
14	Human migration/	7724
15	Minority Groups/	15,995
16	"racial and ethnic groups"/	13,686
17	Cultural Diversity/ or Ethnic Identity/ or Racial Identity/	19,567
18	(immigra* or migrant* or ethnic* or racial* or minorit* or refugee* or non western*).tw	202,447
19	12 or 13 or 14 or 15 or 16 or 17 or 18	212,333
20	11 and 19	7883
21	limit 20 to ("380 aged < age 65 yrs and older > " or "390 very old < age 85 yrs and older > ")	926
22	dementia/	36,553
23	Nursing Homes/	8952
24	exp Nursing Home Residents/	2599
25	older adulthood/	7050
26	Geriatrics/	11,845
27	geriatric psychotherapy/ or geriatric psychiatry/	2126
28	elder care/ or adult day care/	5324
29	((aged or old*) adj2 (people or women or person* or men or immigrant*)).tw	63,217
30	(senior* or geriatric* or elder* or dementia or nursing home*).tw	172,221
31	old*.ti	70,222
32	22 or 23 or 24 or 25 or 26 or 27 or 28 or 29 or 30 or 31	265,295
33	20 and 32	708
34	21 or 33	1318
35	culture change/ or acculturation/ or culture shock/ or multiculturalism/	18,987
36	Identity Crisis/ or Cultural Identity/ or Ethnic Identity/ or Racial Identity/ or Social Identity/ or Group Identity/	31,776
37	cultural diversity/ or cross cultural differences/ or cultural sensitivity/	60,157
38	social identity/ or self-concept/	55,647
39	life satisfaction/ or "quality of life"/ or well being/	93,879
40	(assimila* or accultura* or integrat* or cultural* or culture* or identit* or sociocultural* or multicultural*).tw	662,829
41	(ritual* or tradition*).tw	184,055
42	((meal* or food or eating) adj2 (meaning* or habit* or context* or practice*)).tw	4784
43	(quality of life or life quality).tw	75,916
44	(wellbeing* or well being* or wellness).tw	109,380
45	((life or personal*) adj satisf*).tw	16,207
46	35 or 36 or 37 or 38 or 39 or 40 or 41 or 42 or 43 or 44 or 45	986,318
47	34 and 46	568
48	(food* or eat* or diet* or nutrit* or meal* or cooking or dining).ti	60,299
49	(immigrant* or migrant* or ethnic* or racial* or minorit* or refugee* or non western*).ti	59,647
50	(senior* or geriatric* or elder* or dementia or nursing home* or old* or aged).ti	149,830
51	48 and 49 and 50	31
52	47 or 51	589

Database: Web of Science Indexes = SCI-EXPANDED, SSCI, A&HCI, ESCI Timespan = 1987–2021 Dato: 12.05.2021 Results:853		
#	Searches	Results
1	TS = (food* or eat* or diet* or nutrit* or meal* or cooking or dining)	1,872,467
2	TS = (immigra* or migrant* or ethnic* or racial* or minorit* or refugee* or "non western*")	519,946
3	TS = ((aged or old*) NEAR/1 (people or women or person* or men or immigrant*))	453,085
4	TS = (senior* or geriatric* or elder* or dementia or "nursing home*")	555,294
5	TI = (old*)	291,678
6	#5 OR #4 OR #3	1,148,904
7	TS = (assimila* or accultura* or integrat* or cultural* or culture* or identit* or sociocultural* or multicultural*)	3,442,701
8	TS = (ritual* or tradition*)	954,590
9	TS = ((meal* or food or eating) NEAR/1 (meaning* or habit* or context* or practice*))	21,532
10	TS = ("quality of life" or "life quality")	417,626
11	TS = (wellbeing* or "well being*" or wellness)	162,418
12	TS = ((life or personal*) NEAR/0 satisf*)	43,428
13	#12 OR #11 OR #10 OR #9 OR #8 OR #7	4,755,281
14	#13 AND #6 AND #2 AND #1	699
15	TI = (food* or eat* or diet* or nutrit* or meal* or cooking or dining)	638,225
16	TI = (immigrant* or migrant* or ethnic* or racial* or minorit* or refugee* or "non western*")	168,449
17	TI = (senior* or geriatric* or elder* or dementia or "nursing home*" or old* or aged)	997,790
18	#17 AND #16 AND #15	168
19	#18 OR #14	853

Database: Cinahl Dato: 12.05.2021 Results: 387		
#	Query	Results
S1	(MH "Food")	14,646
S2	(MH "Nutrition")	28,905
S3	(MH "Geriatric Nutrition")	2,682
S4	(MH "Diet")	58,079
S5	(MH "Eating Behavior")	17,792
S6	(MH "Food Habits") OR (MH "Food Preferences")	19,771
S7	(MH "Geriatric Nutritional Physiology")	24
S8	(MH "Meal Preparation")	2,418
S9	(MH "Meals + ")	10,000
S10	(MH "Cooking")	8,373
S11	TI (food* or eat* or diet* or nutrit* or meal* or cooking or dining) OR AB (food* or eat* or diet* or nutrit* or meal* or cooking or dining)	321,863
S12	S1 OR S2 OR S3 OR S4 OR S5 OR S6 OR S7 OR S8 OR S9 OR S10 OR S11	367,250
S13	(MH "Immigrants") OR (MH "Undocumented Immigrants") OR (MH "Refugees")	22,855
S14	(MH "Emigration and Immigration")	6,805
S15	(MH "Transients and Migrants")	5,002
S16	(MH "Ethnic Groups")	29,046
S17	(MH "Minority Groups")	12,517
S18	(MH "Cultural Diversity")	14,020
S19	TI (immigra* or migrant* or ethnic* or racial* or minorit* or refugee* or “non western*”) OR AB (immigra* or migrant* or ethnic* or racial* or minorit* or refugee* or “non western*”)	119,739
S20	S13 OR S14 OR S15 OR S16 OR S17 OR S18 OR S19	155,499
S21	(MH "Gerontologic Care") OR (MH "Geriatric Nutrition") OR (MH "Geriatric Nutritional Physiology")	26,179
S22	(MH "Nursing Homes")	24,579
S23	(MH "Nursing Home Patients")	14,394
S24	(MH "Dementia")	41,999
S25	(MH "Dementia Patients")	2,035
S26	(MH "Senior Centers")	101
S27	TI ( ((aged or old*) N1 (people or women or person* or men or immigrant*))) OR AB ( ((aged or old*) N1 (people or women or person* or men or immigrant*)))	90,444
S28	TI ( (senior* or geriatric* or elder* or dementia or "nursing home*")) OR AB ( (senior* or geriatric* or elder* or dementia or "nursing home*"))	207,725
S29	TI old*	100,658
S30	S21 OR S22 OR S23 OR S24 OR S25 OR S26 OR S27 OR S28 OR S29	368,841
S31	S12 AND S20 AND S30	1,143
S32	(MH "Cultural Values") OR (MH "Cultural Safety") OR (MH "Cultural Diversity") OR (MH "Acculturation") OR (MH "Culture") OR (MH "Food Habits") OR (MH "Food Preferences")	75,111
S33	(MH "Social Identity")	9,558
S34	(MH "Self Concept")	33,004
S35	(MH "Psychological Well-Being") OR (MH "Well-Being (Iowa NOC)") OR (MH "Spiritual Well-Being (Iowa NOC)") OR (MH "Psychological Well-Being (Iowa NOC)") OR (MH "Quality of Life (Iowa NOC)") OR (MH "Quality of Life") OR (MH "Wellness")	148,336
S36	TI (assimila* or accultura* or integrat* or cultural* or culture* or identit* or sociocultural* or multicultural*) OR AB (assimila* or accultura* or integrat* or cultural* or culture* or identit* or sociocultural* or multicultural*)	310,971
S37	TI ( ((meal* or food or eating) N1 (meaning* or habit* or prefer* or context* or practice*)) OR AB ( ((meal* or food or eating) N1 (meaning* or habit* or prefer* or context* or practice*))	6,781
S38	TI ( (“quality of life” or “life quality”)) OR AB ( (“quality of life” or “life quality”))	129,970
S39	TI ( ((life or personal*) N0 satisf*)) OR AB ( ((life or personal*) N0 satisf*))	5,900
S40	s32 or s33 or s34 or s35 or S36 OR S37 OR S38 OR S39	573,207
S41	S31 AND S40	342
S42	TI (food* or eat* or diet* or nutrit* or meal* or cooking or dining)	161,136
S43	TI (immigrant* or migrant* or ethnic* or racial* or minorit* or refugee* or “non western*”)	43,228
S44	TI (senior* or geriatric* or elder* or dementia or “nursing home*” or old* or aged)	233,079
S45	S42 AND S43 AND S44	57
S46	S41 OR S45	387

Database: SocIndex via Ebscohost Dato: 12.05.2021 Results: 450		
#	Query	Results
S1	DE "FOOD habits"	1,480
S2	DE "FOOD consumption"	740
S3	DE "FOOD laws"	25
S4	DE "FOOD & culture"	84
S5	DE "FOOD habits – Social aspects"	42
S6	DE "FAMILY meals"	50
S7	DE "NUTRITION" OR DE "NUTRITION – Social aspects" OR DE "NUTRITION – Psychological aspects"	2,489
S8	TI (food* or eat* or diet* or nutrit* or meal* or cooking or dining) OR AB ((food* or eat* or diet* or nutrit* or meal* or cooking or dining) OR KW ((food* or eat* or diet* or nutrit* or meal* or cooking or dining) OR SU (food* or eat* or diet* or nutrit* or meal* or cooking or dining)	50,533
S9	S1 OR S2 OR S3 OR S4 OR S5 OR S6 OR S7 OR S8	50,533
S10	DE "IMMIGRANTS"	19,884
S11	DE "IMMIGRANTS – Medical care"	72
S12	DE "UNDOCUMENTED immigrants"	1,117
S13	DE "UNDOCUMENTED immigrants – Medical care"	15
S14	DE "OLDER immigrants" OR DE "MINORITY older people"	192
S15	DE "WOMEN immigrants"	903
S16	DE "EMIGRATION & immigration"	23,245
S17	DE "REFUGEES"	6,171
S18	DE "WOMEN refugees"	279
S19	DE "MEDICAL care of refugees"	17
S20	DE "MINORITIES"	10,073
S21	DE "ETHNIC groups"	13,868
S22	DE "ETHNIC differences"	437
S23	DE "CROSS-cultural differences"	2,490
S24	DE "RACIAL differences"	2,276
S25	DE "RACIAL minorities"	403
S26	TI (immigra* or migrant* or ethnic* or racial* or minorit* or refugee* or “non western*”) OR AB (immigra* or migrant* or ethnic* or racial* or minorit* or refugee* or “non western*”) OR KW (immigra* or migrant* or ethnic* or racial* or minorit* or refugee* or “non western*”) OR SU (immigra* or migrant* or ethnic* or racial* or minorit* or refugee* or “non western*”)	224,847
S27	S10 OR S11 OR S12 OR S13 OR S14 OR S15 OR S16 OR S17 OR S18 OR S19 OR S20 OR S21 OR S22 OR S23 OR S24 OR S25 OR S26	226,421
S28	DE "OLD age – Social aspects" OR DE "OLDER women" OR DE "OLDER men" OR DE "SENILE dementia" OR DE "OLDER parents" OR DE "AGING parents" OR DE "MINORITY older people" OR DE "OLDER immigrants" OR DE "OLDER people" OR DE "CROSS cultural studies on older people" OR DE "OLD age"	15,969
S29	TI ( ((aged or old*) N1 (people or women or person* or men or immigrant*))) OR AB ( ((aged or old*) N1 (people or women or person* or men or immigrant*))) OR KW ( ((aged or old*) N1 (people or women or person* or men or immigrant*))) OR SU ( ((aged or old*) N1 (people or women or person* or men or immigrant*)))	43,215
S30	TI ( (senior* or geriatric* or elder* or dementia or "nursing home*")) OR AB ( (senior* or geriatric* or elder* or dementia or "nursing home*")) OR KW ( (senior* or geriatric* or elder* or dementia or "nursing home*")) OR SU ( (senior* or geriatric* or elder* or dementia or "nursing home*"))	63,221
S31	TI old* OR KW old*	34,405
S32	S28 OR S29 OR S30 OR S31	104,156
S33	S9 AND S27 AND S32	450

**Database:** ASSIA (via ProQuest) Advanced search

**Dato:** 12.05.2021

**Results:** 294

noft((food* OR eat* OR diet* OR nutrit* OR meal* OR cooking OR dining)) AND noft((immigra* OR migrant* OR ethnic* OR racial* OR minorit* OR refugee* OR "non western*")) AND (noft(((aged OR old*) NEAR/1 (people OR women OR person* OR men OR immigrant*))) OR noft((senior* OR geriatric* OR elder* OR dementia OR "nursing home*")) OR ti(old*))

**Database:** Social Care Online (Advanced search)

**Dato:** 12.05.21

**Results:** 101

- SubjectTerms:'"older people"' including this term only

- OR SubjectTerms:'"dementia"' including this term only

- OR SubjectTerms:'"nursing homes"' including this term only

- OR AllFields:'old*'

- OR AllFields:'aged'

- OR AllFields:'elder*'

- OR AllFields:'senior*'

- OR AllFields:'geriatric*'

- OR AllFields:'dementia'

AND

- SubjectTerms:'"immigrants"' including this term only

- OR SubjectTerms:'"migrants"' including this term only

- OR SubjectTerms:'"refugees"' including this term only

- OR SubjectTerms:'"immigration"' including this term only

- OR SubjectTerms:'"black and minority ethnic people"' including this term only

- OR AllFields:'immigra*'

- OR AllFields:'migrant*'

- OR AllFields:'ethnic*'

- OR AllFields:'minorit*'

- OR AllFields:'refugee*'

- OR AllFields:'racial*'

AND

- SubjectTerms:'"nutrition"' including this term only

- OR AllFields:'food*'

- OR AllFields:'eat*'

- OR AllFields:'diet*'

- OR AllFields:'nutrit*'

- OR AllFields:'meal*'

- OR AllFields:'cooking'

- OR AllFields:'dining'

To ensure that relevant studies would not be missed, no publication date, country where the study was conducted and restrictions on the methodology were imposed. To locate additional studies, mail notifications were created in the databases. The reference lists of the identified studies were also screened to ensure that relevant research was not overlooked.

### Eligibility Criteria

Peer-reviewed primary studies—regardless of methodology—that provided relevant information about the aim of the current review were eligible for inclusion.

#### Inclusion Criteria

- Studies reporting sociocultural aspects of the food habits/meals/nutrition of older immigrants
- Studies including and specifically present results from participants aged 65 years or older and who are defined as immigrants
- Studies that present subgroups of results or specific statements from participants aged 65 years or older
- Studies published in English
- Studies published all over the world with no limit for publication year or ethnicity for the older immigrants

#### Exclusion Criteria

- Studies including non-immigrant participants but indigenous populations such as Australian aboriginal people, Native Americans and the Sammi population in Nordic countries
- Studies referring to nutrition advice, general recommendations
- Studies describing nutrition experiments/interventions
- Studies focusing on disease-related malnutrition
- Studies focusing mainly on healthy or unhealthy eating behaviour
- Editorials, posters, or conference papers
- Grey literature (reports, policy literature, newsletters, government documents, speeches, white papers)

### Search Outcomes and Study Selection

The references provided by the literature search were exported to EndNote library [25]. After removing duplicates, the references were transferred to Covidence literature screening software [26] for deduplication to allow for a streamlined review process and blinded double screening in all study phases. After the initial search, mail notifications about new articles were created. Additionally, reference lists of the received titles were screened. No studies from mail notifications or from reference lists met the inclusion criteria.

**Fig. 1:**
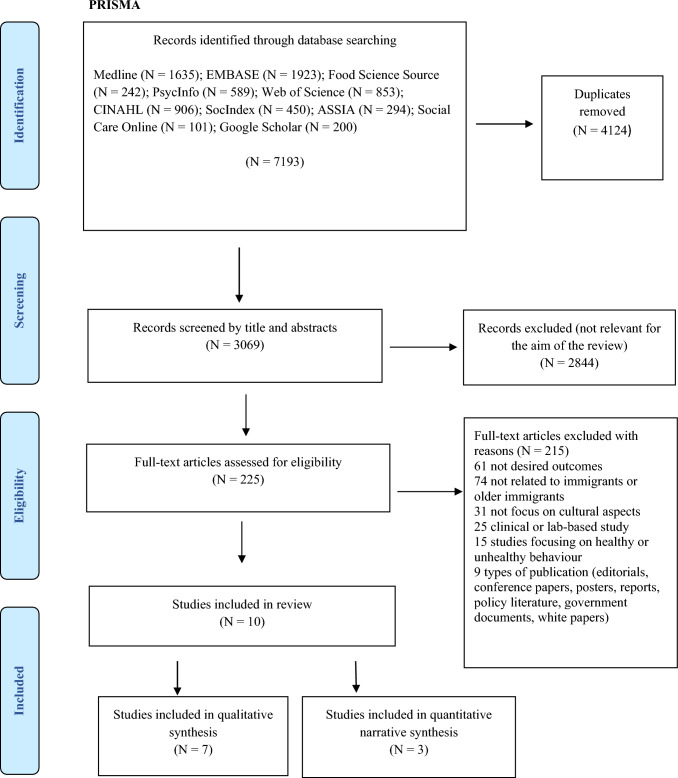
PRISMA flow chart [23]

For study selection, studies were screened in three stages: (i) screening title, (ii) reading abstracts and (iii) reading full-text articles and data extraction. In stage one (i), all authors independently screened a total of 3,069 titles for relevance to the review’s aim. A total of 2,844 records were excluded because they were books, book chapters, conference papers, posters, editorials, PhD theses or protocols or did not meet inclusion criteria. In stage two (ii), the remaining 225 studies were equally distributed between reviewer pairs, with each reviewer pair screening an average of 90 titles and abstracts against the eligibility criteria. At this stage, 215 studies were further excluded. In the last stage (iii), all authors read the full text of the potentially relevant studies and then independently decided whether a study would proceed to data extraction. Discrepancies between reviewer pairs were resolved through discussion until a consensus had been reached or by involving all authors if the first ones failed to settle discrepancies. The search results and reasons for excluding studies are presented in the PRISMA flow diagram (Fig. 1), leaving 10 studies to be included in this review.

### Quality Appraisal

The Mixed Methods Appraisal Tool (MMAT) [27] was employed as a checklist to critically evaluate the studies included in the review. Pairs of two reviewers conducted a quality appraisal of the studies. To avoid erroneous conclusions, uncertainties were solved by reviewers discussing them with each other until consensus was achieved.

The MMAT integrates the quality appraisal of different study designs, hence allowing for an assessment of the methodological quality of multiple methods of studies. The MMAT includes a total of 25 criteria and two screening questions and can appraise five different categories of study designs: (a) qualitative, (b) randomised controlled, (c) nonrandomised, (d) quantitative descriptive and (e) mixed methods. Each category has five core quality criteria rated as ‘yes’, ‘no’ or ‘can’t tell’ (Table 2).

**Table 2:** Quality evaluation of the studies included in review (MMAT-version 2018) [27]

a). Quality evaluation of the qualitative studies included in review					
Study ID/ Screening questions for methodological quality criteria	Is the qualitative approach appropriate to answer the research question?	Are the qualitative data collection methods adequate to address the research question?	Are the findings adequately derived from the data?	Is the interpretation of results sufficiently substantiated by data?	Is there coherence between qualitative data sources, collection, analysis and interpretation?
Porreca, F.I., Unsain, R.A.F., Carriero, M.R., De Morais Sato, P., Dimitrov Ulian, M., & Scagliusi, F.B. (2020) [9], Brazil	Yes	Yes	Yes	Yes	Yes
Girard, A. & Mabchour, A. El (2019) [29], Canada	Yes	Yes	Yes	Yes	Yes
Wu, S. & Barker, J.C. (2008) [30], USA	Yes	Yes	Yes	Yes	Yes
Asamane,E.A., Greig, C.A., Aunger, J.A. & Thomson, J.L. (2019) [32], UK	Yes	Yes	Yes	Yes	Yes
Lam, Y.T.Y, & Keller, H. H. (2015) [33], Canada	Yes	Yes	Yes	Yes	Yes
Lee, S.D., Kellow, N.J., Huggins, C.E., & Choi, T.S.T. (2022) [34], Australia	Yes	Yes	Yes	Yes	Yes
Osei-Kwasi, H.A., Powell, K., Nicolaou, M., & Holdsworth, M. (2017) [35], UK	Yes	Yes	Yes	Yes	Yes

b). Quality evaluation of the quantitative non-randomized studies included in review					
Study ID/ Screening questions for methodological quality criteria	Are the participants representative of the target population?	Are measurements appropriate regarding both the outcome and intervention (or exposure)?	Are there complete outcome data?	Are the confounders accounted for in the design and analysis?	During the study period, is the intervention administered (or exposure occurred) as intended?
den Hartog, A.P., Ramsaransing, G., van der Heijden, L., & van Staveren, W.A. (1996) [36], The Netherlands	Yes	Yes	Yes	Yes	CT
Koochek, A., Mirmiran, P., Sundquist, K., Hosseini, F., Azizi, T., Moeini, A.S., Johansson, S.-E., Karlström, B., Azizi, F., & Sundquist, J. (2011) [37], Sweden	Yes	Yes	Yes	Yes	CT
Tong, A. (1991) [38], USA	Yes	Yes	Yes	Yes	CT

27. Hong et al. The Mixed Methods Appraisal Tool (MMAT) version 2018 for information professionals and researchers. Educ. Inf. 2018; 34: 285–91. https://doi.org/10.3233/EFI-180221

### Data Extraction

A data extraction form was developed based on the aim of the review, including information about the study. The extracted data of the included studies covered the following: (a) author(s), year of publication and country where the study was conducted, (b) title, (c) aim and research design, (d) sample, (e) data collection methods and analysis, (f) findings/results and (g) conclusions. An overview of the studies’ characteristics is presented in Table 3.

**Table 3:** Characteristics of included studies

Author(s) (year) [ref. number], country	Title	Aim	Research design	Sample	Data collection methods	Findings/results	Conclusions
Porreca, F.I., Unsain, R.A.F., Carriero, M.R., De Morais Sato, P., Dimitrov Ulian, M., & Scagliusi, F.B. (2020) [9], Brazil	Dialogues and Tensions in the Eating Habits of Syrian Refugees Living in São Paulo, Brazil	To investigate the processes of acculturation, interculturality and interactions in the eating habits of Syrian refugees who had a Syrian food venue in the city of São Paulo, Brazil	Qualitative study with ethnographic design	N = 10 (Seven cases with ten participants N = 6 males, N = 4 females) were interviewed. When both members of a couple were responsible for the food preparation/ meals, both were interviewed). The age of the participants ranged from 22 to 72. Only one participant aged 65 +	Semi-structured interviews, as well as participant and non-participant observation	The findings demonstrate changes in the preparation of consumed and served food products. These changes were accompanied by tensions, connected to acculturation strategies and intercultural relations. Even with these changes, consuming Syrian food allowed the participants to maintain connections with their original country, despite the sudden temporal and spatial separation	There were changes in the preparation of consumed and served food products. These changes were accompanied by tensions, connected to acculturation strategies and intercultural relations. Even with these changes, consuming Syrian food allowed our participants to maintain connections with their original country, despite the sudden temporal and spatial separation
Girard, A. & Mabchour, A. El (2019) [29], Canada	Meal context and food offering in Quebec public nursing homes: the perspectives of first-generation immigrant residents, family members, and frontline care aides	To gain a better understanding of the meal context and the food offering in Quebec public nursing homes for non-autonomous seniors, particularly with respect to first generation immigrants	Focused ethnography	A total of 26 non-Quebec-born residents. Mean age of participants was about 72 years (N = 13 females and N = 13 males) and their families (N = 24) and frontline care staff (N = 51)	Semi-directed focus groups with six participants on average per group using semi-structured interviews and structured non-participative observations	First generation immigrants arrived in Quebec adapted with difficulty and often not at all to the food offering. Residents’ appetite for food offer was a problem for reasons related primarily to food quality, mealtime schedules, medication intake, physical and mental condition, and adaptation to institutional life. Family/friends often brought in food. Care staff tasks were becoming increasingly tedious and routinized, impacting quality of care	A number of factors complicate how food is prepared in nursing homes, how it is served, and how (where and when) it is consumed by residents. The current food offering is not adapted for immigrant residents; hence, it does not fall within their gustatory frame of reference and, more broadly, within their food culture, which, gives meaning to food and defines what food is “good for us” or “good for me.” Many of these residents do not get used to the food offered and often reject the dishes proposed on the menu. The general living conditions in nursing homes and organizational constraints redefine the aesthesia of meals compared to what residents enjoyed in the community. This has a major impact on the taste, smell, and texture of dishes and on their presentation and the context in which they are consumed. Adapting to these new realities is hard and can take a more or less long time for a fair share of residents and, more specifically, for those from an immigrant background
Wu, S. & Barker, J.C. (2008) [30], USA	Hot Tea and Juk. The Institutional meaning of food for Chinese Elders in an American Nursing Home	To describe how Chinese elders experience their food and mealtimes in nursing homes, and which values, whether Western biomedical or Chinese cultural-are important in how they interpret nursing home food and meals	Qualitative study	N = 33 participants (Seven residents, five women and two men, nine family members, 17 staff members) The mean age of the residents was 81 (ranging from 60 to 91)	20 meal observations and interviews	The participants described institutional food and meals as individualized, nutritious therapy for medical illnesses. Mealtimes lacked sociability and sharing, and although family members provided Chinese food, they did not eat with residents. Residents generally did not consider the institution’s effort to provide an “Asian diet” of hot tea and *juk* (rice porridge) to be Chinese food. These findings suggest that, for these Chinese elders, the bio-medicalised, individualized food service and mealtime caregiving practices stripped food of its meaning as a social, shared mealtime experience with family	Adequately addressing the need for cultural competence in food service requires assessing to what extent both the kinds of food and the way it is served is important for residents. Although the study findings suggest that family-style meals might foster sociability and friendship among residents that does not now exist, the existing regulatory structure, the need for efficient production (in contrast to labour-intensive Chinese cooking), medical individualization, hygienic food service, and safety-oriented caregiving, must be reconciled with a communal style of eating. Daily monitoring of intake would be more difficult because portions would not be individualized on a tray. However, longer term (e.g., weekly) monitoring of weight and other signs of nutritional status could still be performed, as could monitoring of other aspects of psychosocial well-being, such as social interaction levels
Asamane, E.A., Greig, C.A., Aunger, J.A. & Thomson, J.L (2019) [32], UK	Perceptions and Factors Influencing Eating Behaviours and Physical Function in Community-Dwelling Ethnically Diverse Older Adults: A Longitudinal Qualitative Study	To: (1) identify and compare factors influencing eating behaviours and physical function among ethnic older minorities living in Birmingham, United Kingdom; and (2) understand how these factors and their association with healthy eating and physical function changed over 8-months	Longitudinal qualitative design	A total of 100 older adults aged 60 years or older living within the Birmingham area who self-identified as African, Indian, Pakistani, Bangladeshi, or Caribbean were recruited using purposive sampling. Baseline (N = 92) and follow-up (N = 81)	Two in-depth qualitative interviews (baseline and 8-month follow-up)	1. The differing perceptions of healthy eating and physical function 2. The personal, social, and cultural/environmental factors influencing eating behaviours and physical function and how these factors differ among the sample 3. Perceived changes to eating behaviours and physical function over the 8-month follow-up period	This longitudinal qualitative study found diverse perceptions of healthy eating and physical function among ethnically diverse older adults. Traditional foods were highly regarded as healthy foods by African and Caribbean older adults. The presence of super-diversity was reported as positively influencing accessibility and affordability of traditional foods. Diversity supported them to feel comfortable and encouraged to shop, eat and engage with other cultures
Lam, Y.T.Y, & Keller, H. H. (2015) [33], Canada	Honoring Identity Through Mealtimes in Chinese Canadian Immigrants	To examine: 1) the meaning of the mealtime experience through six community-dwelling dyads/triads of Chinese Canadian People with Dementia and their family care partners in the Greater Toronto Area, 2) to specifically explore how honouring identity is experienced in this culture 3) to determine whether the Life Nourishment Theory (LNT) needs to be extended due to the findings of this ethnocultural group	Qualitative study	A total of 6 participants Chinese Canadian immigrant, (N = 5 males, and N = 1 female, aged 80–100)	A semi-structured interview guide was used to interview Dyad/triad family and individual interviews	This sub-study provided insight into the challenges and rewards of mealtimes for Chinese immigrant families with dementia in the community and specifically provided further insights into the honouring identity concept. Although Life Nourishment Theory and specifically the honouring identity concept was generally confirmed in this group, some culturally specific themes were also identified	This sub-study provided insight into the challenges and rewards of mealtimes for Chinese immigrant families with dementia in the community and specifically provided further insights into the honouring identity concept. Although Life Nourishment Theory and specifically the honouring identity concept was generally confirmed in this group, some culturally specific themes were also identified
Lee, S.D., Kellow, N.J., Huggins, C.E., & Choi, T.S.T. (2022) [34], Australia	How and Why Diets Change Post-Migration: A Qualitative Exploration of Dietary Acculturation among Recent Chinese Immigrants in Australia	To explore how and why diets change post-migration for Chinese immigrants living in Australia	Qualitative study	A total of 11 participants (N = 3 males and N = 8 females, ranging in age from 22–68 years with length of residence in Australia ranging from 1–8 years). Two participants aged 65 +	Semi-structured interviews	Thematic analysis revealed that participants exhibited changed social structures of meal preparation, and made unacknowledged dietary changes, such as recipe modification, to maintain their traditional Chinese diet post-migration	Diets of Chinese immigrants change post-migration and many of these changes are in relation to the social structures of food preparation. Many aspects of their traditional diet are also maintained, but in a new way that integrates their new lifestyle with their preference for eating Chinese foods. Some purposeful dietary change occurs in relation to breakfast and snacking behaviours while recipe modification of traditional Chinese foods results in unacknowledged dietary changes. Changes towards more convenient, Western-style foods might lead to potentially unhealthy dietary changes in Chinese immigrants, which may contribute to an increased risk of cardiometabolic disease over time. Cultural identity and familiarity remained important factors influencing food choice post-migration and, especially with older migrants, some resistance to change or a longing to maintain Chinese eating habits was evident
Osei-Kwasi, H.A., Powell, K., Nicolaou, M., & Holdsworth, M. (2017) [35], UK	The influence of migration on dietary practices of Ghanaians living in the United Kingdom: a qualitative study	To explore the influence of migration on dietary practices and the process of dietary acculturation amongst Ghanaians living in the UK	Qualitative study	A total of 31 participants (N = 19 women and N = 12 men between 25–68 years of age, of Ghanaian ancestry, living in Greater Manchester). Three participants aged 65 +	Face-to-face interviews	Three distinct dietary practice typologies were discernible that differed in terms of typical meal formats, meal contexts, structure and patterning of meals, food preparation and purchasing behaviours: (i) continuity practices; (ii) flexible practices; and (iii) changed practices. The identified practices were shaped by interrelating factors that fell into four main clusters: social and cultural environment; accessibility of foods; migration context; and food beliefs/ perceptions	Participants retained, to a varying degree, some aspects of Ghanaian dietary practices, whilst adopting key features of UK food culture. This study demonstrates the complexity of dietary change, indicating that it is not a linear process, and it is dependent on several factors
den Hartog, A.P., Ramsaransing, G., van der Heijden, L., & van Staveren, W.A. (1996) [36], The Netherlands	Migration, nutrition, and the elderly. Food habits of the elderly Hindustani women in Utrecht, the Netherlands	To get insight into food habits and nutrition of elderly migrants by taking a groups of Hindustani elderly women as a case study	Case study	N = 44 Hindustani women (average age 65)	Survey	All respondents stated that they were content with their food and nutrition in the Netherlands. Although most respondents were content with their food, 23% said to miss from time-to-time special Surinamese products such as vegetables, fruit and fish. Most women bought their food two or three times a week from a nearby ethnic shop and/or supermarket or from The Hague or Amsterdam because the availability of Surinamese products is more varied The results show that the dietary patterns of the elderly Hindustani women have several elements of the Dutch dietary pattern, particularly the consumption of bread and the habit to put spread on slices of bread: cheese, peanut butter, egg, or sardines. Compared to consumption in Suriname, the respondents stated to consume less rice and other typical Surinamese products were much less consumed. However, the consumption of vegetables has not changed much. Milk and milk products and more meat and chicken were often consumed compared to consumption in Suriname	The changing food habits of the elderly Hindustani women are the result of an adaptation to the new environment and a relative increase in standards of living. Another dimension of change is not only the duration of their stay in the Netherlands, but likewise their place in the life cycle. Elderly people eat differently from the young ones because of other physiological and social needs. The available data suggest that the elderly can reasonably well cope with their food
Koochek, A., Mirmiran, P., Sundquist, K., Hosseini, F., Azizi, T., Moeini, A.S., Johansson, S.-E., Karlström, B., Azizi, F., & Sundquist, J. (2011) [37], Sweden	Dietary differences between elderly Iranians living in Sweden and Iran a cross-sectional comparative study	To examine possible dietary differences between elderly Iranians living in Stockholm, Sweden with elderly Iranians living in Tehran, Iran, taking into account sex, age, marital status, and education	Cross-sectional study	Iranians living in Stockholm (N = 121) Iranians living in Tehran (N = 52), aged 60–80	Survey. Dietary intakes were assessed by semi-quantitative food frequency questionnaire The results were analysed by bootstrapped regression analyses with 1000 replications	Iranians living in Sweden had significantly higher intake of protein, total fat, and fiber than Iranians living in Iran, but lower consumption of carbohydrates. The observed differences in intake of macronutrients were reflected in consumed amount of all food items, which were higher among Iranians living in Iran with the exception of bread and grain consumption which was lower	There are general differences in dietary habits between Iranians living in Iran and Iranians living in Sweden. Parts of observed differences in dietary habits may reflect a favourable adoption process to the Swedish dietary habits after migration. Meanwhile other differences are point of concern in light of the high prevalence of overweight, among Iranians living in Sweden and can have unfavourable impact in particular in the context of cardiovascular health
Tong, A. (1991) [38], USA	Eating Habits of Elderly Vietnamese in the United States	To focus on the eating habits of elderly Vietnamese and the association between their health status and their culturally determined dietary practices and beliefs	Cross-sectional study	A sample of 62 elderly Vietnamese (N = 37 women and N = 25 men, ranging in age from 50 – 89 years). N = 38 participants aged 65 +	Survey and open-ended a 24-h dietary recall interview regarding their eating habits and food preferences in order to examine their health status	The results indicate that their eating habits and cultural food preferences tend to remain similar to when they lived in Vietnam. Their daily meat and fruit consumption, however, appears to have increased	Results indicated that one-fifth of those sampled had milk at breakfast. The majority of the elderly still consumed rice at both lunch and supper, and 94 percent did not snack. Thirty-one percent took vitamin supplements. The health problems most frequently mentioned were anaemia, arthritis, hypertension, and diabetes. Research on food habits and diet in relation to diseases of this ethnic group is much needed

### Data Synthesis

A narrative summary was undertaken for the qualitative studies [28]. The data were summarised under the same categories. Although two of the studies [29, 30] also present findings generated from interviewing healthcare professionals and/or family members, only data generated from older immigrants were extracted, analysed, and included in the synthesis.

Because of the heterogeneity in study designs, the methods for data collection and outcomes of the quantitative studies included in the review, a meta-analysis was not feasible. Therefore, to integrate and analyse the information, a narrative synthesis which adopts a textual approach to summarise the findings of systematic reviews, was performed for these three quantitative studies [31].

## Results

### Study Characteristics

The search for empirical studies resulted in a total of 10 studies: seven qualitative [9, 29, 30, 32–35] and three quantitative, nonrandomised [36–38]. The qualitative studies included in the current review were of good quality, met the MMAT’s assessment criteria and were found to provide sufficient information needed to determine their methodological quality. The quantitative studies described no intervention or measurement; therefore, we could not assess if the intervention was administered (exposure occurred) as intended.

### Year of Publication and Country of Origin

The period of publication of the included studies was between 1991 and 2022, peaking at two articles published in 2019. The studies originated from Australia (1), Brazil (1), Canada (2), The Netherlands (1), Sweden (1), the UK (2) and the US (2). Most of the studies were conducted in Canada, the UK, and the US.

### Aim and Research Design

The aim of the qualitative studies included in the review was to explore older immigrants’ food habits and meal preferences after immigration, how and why diets changed after immigration and the influence of migration on dietary practices and the process of dietary acculturation [33–35]. Two studies explored the meal context and food offered in Canadian and US public nursing homes with respect to first-generation immigrant residents [29, 30]. One study had a longitudinal qualitative design [32], two had ethnographic designs [9, 29], three had qualitative descriptive designs [30, 33, 34], and one had a qualitative explorative design [35]. Among the quantitative studies, one had a case study design [36] while the other two had a cross-sectional design [37, 38]. One study additionally aimed to obtain insights into the possible dietary differences between older Iranian immigrants living in Sweden and older Iranians living in Teheran, Iran [37].

### Sample

Overall, this review presented the perspectives or experiences of 488 participants. The sample in all studies consisted of participants aged 65 and older.

The sample in the qualitative studies consisted of 209 participants (N = 102 women and N = 107 men). The sample in one of the studies [29] consisted of nursing home residents (N = 26), family members (N = 24) and frontline care staff (N = 51), but information about the gender of the participants was provided only for the nursing home residents (N = 13 females; N = 13 males). Besides older immigrants, three qualitative studies also included family members from immigrant families [9, 29, 30] and healthcare personnel [29, 30]. A total of 279 respondents, both women (N = 182) and men (N = 97), participated in the quantitative studies.

Except for one study [29] that referred to study participants as ‘first-generation immigrant residents’ without giving further information about their nationality, the rest of the studies provided complete demographic information about the sample, that is, age, gender, ethnicity, marital status and living conditions, education level, faith/religion, length of residence in the host country, dietary patterns and eating habits before and after immigration. Only two studies [9, 38] provided information about the older immigrants’ status as refugees at the time of immigration.

Eight studies included only one cultural group, as reported by nationality, for example, Iranians [37], Ghanaians [35], Vietnamese [38], Syrians [9], Hindustani [36] or Chinese [30, 33, 34]. One study included more than one ethnic or national group, referring to the participants as ethnically diverse older adults who identified themselves as Africans, Indians, Pakistani, Bangladeshi or Caribbean [32]. In another study, the participants were referred as ‘non-Quebec born residents, all non-native French or English speakers from a wide range of sociocultural and geographic horizons’ [29] (p. 227), providing no further information about their migration history or socioeconomic and cultural characteristics. Although most of the studies included home-dwelling older immigrants, two studies were conducted within the nursing home context, including nursing home residents [29, 30].

### Data Collection and Data Analysis

Among the qualitative studies, four studies employed only in-depth individual interviews using a semi-structured interview guide as the data collection method [32–35]. One study used focus group interviews with six participants on average per group [29]. Three studies also used observation in addition to interviewing during data collection [9, 29, 30]. Thematic analysis [9, 29, 30, 33–35] or content analysis [32] were used to analyse the data generated from interviews, focus groups and observations.

Quantitative studies [36–38] used surveys to collect data. One study also used a 24-h recall dietary recall procedure, here with open-ended questions [38]. Data were analysed using descriptive statistics.

Three studies employed theoretical frameworks when discussing the findings [32, 33, 36].

### Summary of the Findings

Twelve categories underpinned by 22 findings were meta-aggregated into three synthetised findings: (i) the significance of food in maintaining cultural identity; (ii) the continuity of traditional food culture; and (iii) adapting to the host country’s food culture. The synthetised findings, categories and extracted findings are presented in Table 4.

**Table 4:** Synthesised findings and categories and extracted findings

Synthetised findings	Categories	Findings
The significance of food in maintaining cultural identity	Traditional food preferences [35, 38] Diet and nutrition beliefs [30, 38] Food as an expression of culture, identity, and relationships [32, 33] Keeping culture with food ways [33]	Eating known and most familiar staple foods for all ages (rice for Asians) Using food to preserve traditional culture Eating ‘healthier’ because immigrants can access the foods they prefer and value Traditional food satiates for longer Continuity in food eating to uphold traditional individual and family identities Eating traditional food is part of their ‘identity’, ‘heritage’ or ‘culture’ Sharing food and eating together (dim sum in Chinese culture) Food linked to one’s identity, with mealtimes helping create a sense of belonging Food and meal practices mean more than nutrition and are dependent on social context for their meaning Food connects older immigrants to the past (brings good memories for those living with dementia) Recognisable food that represents something good to ‘us’ or ‘for me’
The continuity of traditional food culture	Absence of dietary changes during food preparation [9] Flexibility and recipe modification [34] Availability of and access to traditional food [32, 35] Flavour – the driver of food choice [29, 34, 35] Food shopping practice [36, 38]	Accessibility and affordability to diverse traditional foods help immigrants feel comfortable When possible, using the same recipe and ingredients as used in their country Preferring food with taste (diversity in seasoning, cooking techniques and sauces using distinctive ingredients) instead of host country foods Being content with the availability of traditional food products (exotic fresh fruits and vegetables, fish, etc.) in ethnic shops or supermarkets
Adapting to the host country’s food culture	The length of residence in the host country and immigrants’ age when arriving in the host country [36] Embracing new foods and new mealtime routines [9, 34, 36–38]	The longer they live in the host country, the easier it is to adopt food products specific to the host country Younger immigrants change their food consumption after migration No access to traditional food in nearby food shops Changes in food habits because of a new environment (i.e., moving to a nursing home – institutional practices) Modified dietary habits to cope with changes reflecting economic and living conditions in the host country Changes in family mealtime routines because of lifestyle, work schedule, means of transportation or housing Changes in the way some foods are eaten (a phenomenon seen among children/younger)

### The Significance of Food in Maintaining Cultural Identity

The results of the included studies revealed that most of the immigrants, especially the first-generation immigrants, emphasised strong connections between their cultural food and ethnic identity. The importance of maintaining cultural identity through various cultural, traditional food practices, such as eating certain types of food and the ways food was prepared, was emphasised in several studies [30, 32, 33, 35, 38]. Preparing and eating ‘our food’ or ‘African foods’ [35] or ‘Chinese food’ [30] was important for maintaining ‘identity’, ‘our culture’ as well as a ‘legacy’ that should be passed on to the next generation [33]. For Asians, rice and tea served at almost every meal were staple foods for all ages [30, 38]. Moreover, keeping food routines and traditions, such as going every week to dim sum (restaurants serving traditional Chinese cuisine of bite-sized food served in small steamer baskets) and meeting other Chinese families, supported family’ values, thus honouring identity in Chinese culture [33] Connections between food and identity hinged upon memories evoked through sensory engagement with ethnic food, thus linking ethnic food with memories of home [9], or memories of childhood, as echoed in another study [38]. In addition, eating and retaining traditional food practices was found to uphold traditional individual and family identities and roles [33]; for example, across all cultures, women tend to hold significant food-related roles associated with providing food for their families as wives and mothers.

### The Continuity of Traditional Food Culture

Overall, there were several reasons why certain aspects of diet did not change after immigration. One of them referred to the availability of and accessibility to ethnic food stores in the host country over the past few decades, thus enabling the continuity of original food culture [32, 35, 36]. However, the review also indicated that if a known food was not available, the participants searched in the new food culture for foods similar to those from their original food culture or used the same recipe and ingredients as they used in their country of origin to maintain their cultural eating habits [9]. On the other hand, being flexible and substituting or omitting ingredients in traditional Chinese recipes, for example, when they were not available in the host country was another strategy to perceive the diet as not changing after immigration [34]. Nevertheless, although many modifications in food preparation occurred, some preparations could not be modified to maintain the original taste and flavour [9].

Another reason for continuing to eat traditional food was related to personal taste preferences, such as preferring spicy ingredients [35]. The studies also revealed that immigrants of older age were more likely to maintain their well-established eating habits, which remained similar to those they had when they lived in their country of origin [34, 38]. Although old age and different physical conditions and cognitive impairment lead to older immigrants moving to a nursing home, food preferences remained the same, with the older immigrants expecting to eat food inspired by their cuisine from their country of origin and retain a premigration style of eating [29, 30, 33].

Familiarity with cooking methods, especially within the Chinese food culture [30, 34], was important for the continuity of preparing and eating ethnic foods. However, the findings revealed that immigrants’ personal health beliefs and habits were important drivers to continue preparing and eating foods from their country of origin. For older Chinese immigrants, drinking hot instead of cold water [34], was a habit that was difficult to change. For older Muslims, continuing to eat halal meat, in accordance with Islamic law [9], was also important for keeping eating traditions alive. Moreover, believing that preparing and eating traditional foods from the country of origin was healthier than eating traditional foods from the host country [35] was another reason contributing to the continuity of the original food culture.

### Adapting to the Host Country’s Food Culture

Although the findings revealed that keeping traditional foods was important for immigrants’ identity and their legacy, adopting some elements from the host country’s food culture was also observed [9, 35, 36]. When confronted with a new environment and differences in the availability of food in the host country, several immigrants needed to adapt and change their eating habits. The studies also revealed that older immigrants partially or totally adopted the foods and eating habits from the host country [9, 35, 37]. Most of the changes occurred because of different routines and lifestyles they had in the host country, such as a busy work schedule, means of transportation, housing and family income [9] or because they did not have the necessary time to prepare the meals knowing that the preparation of varied dishes takes time [9, 36]. Another reason that food and eating habits changed, thus leading to dietary acculturation, was related to the perception that foods from the host country were perceived as healthier than foods eaten in their country of origin [37].

Changes in food and eating habits after immigration were also related to the immigrants’ age at the time of immigration. Those studies providing data on both the older and new generations showed that younger immigrants more easily adopted food products and food preparation specific to the host country compared with older immigrants [9]. Although the first-generation immigrants still prepared and are foods from their country of origin [33, 34], the second-generation has gradually changed the foods and eating habits specific to the host country, eating original foods from their country of origin only occasionally and in contexts such as family visits or celebrations [35].

Another change that occurred was related to the duration and purpose of their stay in the host country—the longer the period that the immigrants had been residing in the host country, the easier they were able to change their food consumption habits [36].

Although the changes in food preparation and eating habits after immigration were, for most immigrants, voluntary choices, the findings from two studies [29, 30] indicated that growing old in the host country and moving to a nursing home led to involuntary changes in food and eating habits that were mandatory because of institutional practices.

## Discussion

In the current literature review, we identified and synthesised 10 studies addressing older immigrants’ food habits and meal preferences after immigration and settlement in the host country. Our review has revealed that preparing and eating ethnic foods is an important part of everyday eating practices. For older immigrants, food and mealtimes were strongly linked to cultural and ethnic identities. Ethnic food was valued as an important bond connecting them to their country of origin. Moreover, preparing and eating ethnic foods created nostalgia for their country of origin, hence becoming part of remembering their past and acknowledging their heritage, thus providing a sense of belonging.

Maintaining cultural identity through the preparation and eating of ethnic food appeared to be more important to first-generation immigrants, which was especially true for women who had significant food-related roles in earlier life and was associated with providing food for their families as wives and mothers [33]. These feelings were echoed in a study on the meaning of food and food preparation for Goan women in Toronto, Canada, and the role of food in creating and maintaining distinctly gendered ethnic identities [39], revealing that among other things, the power of food and foodwork in transmitting ethnic identity and the opportunity to draw family and community together, thus maintaining collective solidarity and identity. As also pointed out in an early study [40], because of the strong connection between food and identity, immigrants tend to conserve their eating habits. For them, food is one of the remaining bonds with the country of origin and a strong and meaningful component of their identity, being a prominent feature for elderly Vietnamese [38] and Chinese [33] living overseas.

Our review also found that identity is preserved through food practices and sharing [32]. Chinese immigrants displayed their ethnic identities and reinforced their bonds to others who claimed the same heritage through the tangible medium of food. By sharing foods that they perceived to be traditional with other Chinese, they were reinforcing their common bond, even in a diaspora [33]. These findings were similar to the findings presented in a study conducted among Barbadian immigrants in Atlanta, US, showing that Barbadians consciously deployed conspicuously Barbadian foods to signify their ethnic and national identity, both in situations where they were demonstrating solidarity with the Barbadian community, as well as to distinguish themselves as distinctly Caribbean in mixed American settings [41].

For older immigrants living in a nursing home, when the institutional food did not meet the residents’ characteristics in terms of immigrant background and key elements of their cuisine, particularly seasoning and cooking techniques, family and friends chose to bring in home-cooked food that the resident appreciated and ate with gusto [29], thus honouring individual and family identity, which was special for those living with dementia [33]. Moreover, serving ‘recognisable’ dishes brought by family members has been shown to allow older immigrants living in nursing homes to maintain and reaffirm their ethnic identities, especially for those living with dementia. The idea that traditional food strengthens the feelings of belonging, identity and heritage, thus helping persons with dementia hold on to and reinforce their cultural identity, was also supported by the findings presented in a Norwegian study revealing that familiar tastes and smells awoke pleasant memories in people with dementia and boosted their sense of well-being, identity and belonging, even producing words in those who usually did not speak [42]. Moreover, food could bring back memories of childhood that reflected the usual practices and traditions that continue to influence current preferences.

The results from a recently conducted scoping review [18], showed that immigrants experienced that there was an overall abundance of food in the host country, with traditional foods being always available, not just seasonally as the immigrants were used to. The influx of ethnic minority corner shops and supermarkets in communities and larger cities, making available fruits, vegetables and spices, reinforced continuity of traditional food habits and meal preferences. This accessibility and affordability of traditional foods contributed to the continuity of the traditional food culture in older immigrants, which is in line with findings from our review [32, 35, 36]

The importance of spices as markers for the smell and flavour of food, thus influencing food choices, emerged in several qualitative studies [9, 29, 34, 35]. This idea was supported by findings from other studies [14, 43], suggesting that spicy food preparation was more valued and essential for older immigrants to preserve the original food culture after immigration and could not be replaced by commercially available food items. Our review has also shown that first-generation immigrants attempted to preserve food habits perceived as being of their country of origin, including spiciness in food [9, 34, 35]. This was the most visible in the studies with a quantitative design where food preferences and type of foods eaten before and after immigration were displayed and compared with the majority population from the host country [36, 38] or with the same ethnic group living in their country of origin, showing noticeable differences in food habits and meal preferences between older immigrants living in the host country and those living in their country of origin [37].

Sometimes, to preserve food habits and meal preferences, flexibility, and recipe modification were needed [34]. Moreover, unfamiliar ingredients were adapted to preserve known food taste by adding spices [9]. Flavours and distinct tastes are the strongest characteristics of traditional food [44]; therefore, our review indicates that, to preserve traditional familial food, older Chinese immigrants used mealtime as learning opportunities to teach younger generations cultural and traditional principles of food practices [33].

Although the significance of eating traditional food in maintaining older immigrants’ cultural identity was emphasised, similar to findings from another systematic review [45], our review has revealed a process of change in food habits and meal preferences among older immigrants [36]. Some of these changes occurred because of the older immigrants’ perceptions of the host country’s foods being healthier than their own traditional food [37]. Because of altered routines and lifestyles with a busy work schedule and lack of time to prepare traditional foods [9, 35], food changes were unavoidable. Other changes occurred involuntarily because of the process of ageism and altered sense of taste affecting their usual food habits, hence avoiding certain cultural spicy foods [30, 33]. For older immigrants living in nursing homes, the lack of culturally recognisable mealtimes contributed to altering their food habits [30]. Moreover, changes in their food habits consisted not only of adopting different foods from the host country or adapting to new food flavours, but also of changing the number of meals they consumed in the day, usually skipping breakfast and/or lunch [9, 34, 37]. However, eating traditional foods became a feature that occurred only on occasions such as family visits or feasts [35].

Accessibility and availability of ethnic food shops contributed to immigrants who migrated to the Western parts of the world in the last decade not perceiving challenges with maintaining their cultural food habits and meal preferences. In contrast, because of a lack of availability and accessibility to traditional foods, immigrants who migrated in the early 1970–1980s and grew old in the host country had to adopt certain foods [35] and dietary patterns [36, 37]. This contradicts the findings from a previous study [46], stating that, in the UK, the older generations of South Asians were less likely to change their food habits since they were more segregated from the majority population, thus continuing to eat traditional foods. This supports Kocktürk-Runefors’ [47] theory suggesting that staple foods are the last to change following the postimmigration period. In contrast, younger generations of South Asians were found to be more likely to change their food habits and meal preferences by including English foods in their daily meals [46]. These findings are in line with the findings from our review, where the younger generation accepted the host country’s foods easier than the older generation from the same family [9]. However, our review suggests that not only did the younger generation adopt the host country’s foods, but for several reasons, older immigrants living in the host country did as well [34–37]. For example, Chinese immigrants have often adopted a bicultural eating pattern, appearing to be caught at the intersection of East and West food cultures [34] and, thus, preserving elements from their Chinese culture and adopting some elements from the host country. This contrasts the findings from a previous study reporting that Chinese immigrants minimally changed their food habits, even as time spent living in the UK increased [48].

An important factor influencing the adoption of new foods was the age of immigrants at the time of immigration [36]. Our review revealed that the older immigrants were at the time of immigration, the more difficult it was for them to change their food habits and be open to dietary acculturation. However, according to Cleveland et al. [49], acculturation is a complex process based on assimilation of the new culture (majority, the host) and the preservation and promotion of the culture of origin (minority), here further referring to Berry’s [11] four patterns of acculturation: integration, separation, assimilation and marginalisation, where integration is the most common applied pattern—adopting the new culture and, at the same time, cultivating their culture of origin. This supports the findings from our review.

The length of residence in the host country was found to influence the degree to which immigrants adopted the host country’s food habits. As our review suggested, the longer the stay, the easier it was for immigrants to adopt the host country’s food habits and meal preferences; these findings are supported by the results from a study conducted among Romanians living in Andalusia, Spain [13].

Finally, when people immigrate and settle dawn in a new country, in their ‘migrant suitcase’ they not only bring their food with them, but also their representations and ideals of good food [43]. Therefore, for older immigrants, eating ‘food from home’ can reconnect them with a sense of home, hence contributing to improving their quality of life in the host country.

## Strengths and Limitations

To the best of our knowledge, this is the first review to present older immigrants’ experiences with food habits and meal preferences after immigration and settlement in the host country. We sought studies that included diverse groups of immigrants with different designs and methods of data collection. Several studies included Chinese immigrants in Australia, Canada, the UK, and the US – countries well-known for their increased level of immigration [50, 51], hence to some degree reducing the transferability of the findings.

The included studies revealed older immigrants’ experiences with food habits and meal preferences of both men and women from different ethnic groups belonging to several countries around the world, hence increasing the diversity with respect to ethnicity, gender and age. The participants in the included studies were home-dwelling older immigrants, but also those living in nursing homes. Because of the paucity of research focusing on older immigrants’ food habits and meal preferences, we also included studies presenting subgroups of results or specific statements from participants aged 65 years or older. In some studies, the sample was also comprised of family caregivers and/or healthcare personnel. To avoid bias during the data synthesis, only data generated from the participants defined as older immigrants were analysed and further synthesised. Statements from family caregivers or healthcare personnel were not included in the synthesis.

We are aware that, because of the search strategy, some relevant literature may have been missed, though we did not limit the period of published research to a certain period. Because of the aim of the review, we have not mentioned the problem of a relatively high prevalence of noncommunicable diseases, such as diabetes, heart diseases or obesity, among older immigrants. Major barriers to healthy eating among older immigrants included lack of nutrition knowledge, families’ preferences, cultural values, religious dietary proscriptions, financial constraints and low availability of food quality. These issues require further research.

## Implications for Research and Practice

The knowledge from the current review has several implications for health and social services that should be considered. An ageing immigrant population will gradually be challenged with difficulties in coping with their food and nutrition, besides potential other health challenges; therefore, one may wonder who will take care of these individuals when they face these challenges. With family ties becoming loser and attitudes towards family caregiving constantly changing [21], public health and social services play an important role in providing adequate cultural nutritional care to older immigrants. Healthcare personnel should be aware of the food and eating habits of older immigrants. It is important that healthcare personnel have competence and opportunities to meet the needs of culturally appropriate food habits of older immigrants. Not acknowledging older immigrants’ food habits and meal preferences could be a source of stress for this population when they are staying in a hospital or nursing home. Therefore, this issue should be carefully addressed by healthcare and social services and in future research.

## Conclusions

Although there is a broad range of literature on food, immigrants and migration, this topic has not been fully investigated regarding older immigrants. There is a paucity of research focusing on older immigrants’ food habits and meal preferences in the postimmigration period and the role food plays in shaping a sense of belonging among this group. However, our review revealed that older immigrants, regardless of being newly arrived or belonging to those who aged in the host country, have different food habits and meal preferences compared with the majority population in the host country. Depending on several factors, such as availability and accessibility of ethnic food shops, the age immigrants came to the host country, the length of their stay in the host country and alterations of their lifestyle, different forms of dietary acculturation exist. Despite these differences, our review has revealed that older immigrants wish to maintain their traditional food habits and meal preferences. For them, although they adopted some culinary elements from the host country, eating traditional food contributed to maintaining their cultural identity and to the continuity of traditional food culture.

Healthcare personnel should make efforts to target food literacy skills in older immigrants and encourage them to try new foods and eat healthier. Finally, understanding older immigrants’ food habits and meal preferences helps health and social services ensure culturally appropriate nutritional care, thus optimising their health.

### Supplementary Information

Below is the link to the electronic supplementary material.

Supplementary file1 (DOCX 34 KB)
